# Spin-dependent recombination probed through the dielectric polarizability

**DOI:** 10.1038/ncomms9534

**Published:** 2015-10-06

**Authors:** Sam L. Bayliss, Neil C. Greenham, Richard H. Friend, Hélène Bouchiat, Alexei D Chepelianskii

**Affiliations:** 1 Cavendish Laboratory, University of Cambridge, Cambridge, CB3 0HE, UK; 2 LPS, Université Paris-Sud, CNRS, UMR 8502, F-91405 Orsay, France

## Abstract

Despite residing in an energetically and structurally disordered landscape, the spin degree of freedom remains a robust quantity in organic semiconductor materials due to the weak coupling of spin and orbital states. This enforces spin-selectivity in recombination processes which plays a crucial role in optoelectronic devices, for example, in the spin-dependent recombination of weakly bound electron-hole pairs, or charge-transfer states, which form in a photovoltaic blend. Here, we implement a detection scheme to probe the spin-selective recombination of these states through changes in their dielectric polarizability under magnetic resonance. Using this technique, we access a regime in which the usual mixing of spin-singlet and spin-triplet states due to hyperfine fields is suppressed by microwave driving. We present a quantitative model for this behaviour which allows us to estimate the spin-dependent recombination rate, and draw parallels with the Majorana–Brossel resonances observed in atomic physics experiments.

## Abstract (web summary)

Weak spin-orbit coupling in organic materials results in unique spin-dependent phenomena which may be exploited in spintronic or photovoltaic devices. Here, the authors use the dielectric properties of carbon-based semiconductors to study spin-selective carrier recombination during magnetic resonance.

## Graphical abstract

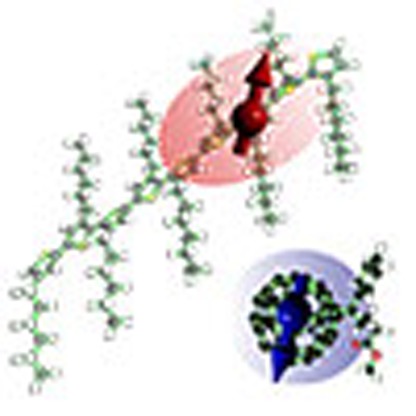

Carbon-based semiconductors give rise to a host of spin-dependent phenomena that directly influence optoelectronic functionality, and open up appealing possibilities in the emerging field of organic spintronics^1^,^2^. Organic photovoltaic devices (OPVs) are a particular example where the role of spin is of critical importance in determining solar cell performance^3^. In these systems, the weak spin-orbit coupling gives rise to strictly obeyed spin-selection rules, making the properties of a photoexcited species vary drastically with its spin configuration. For example, recombination to a spin-singlet (*S*=0) ground state, will only be efficient for excited states that have singlet character. Triplet (*S*=1) excited states will experience much slower recombination rates since spin-selection rules prevent coupling to states with different spin multiplicity. One important case of spin-dependent recombination in organic solar cells is among the weakly bound electron-hole pairs, or charge-transfer (CT) states, that form across the interface between donor and acceptor materials^4^,^5^,^6^,^7^,^8^. Due to the low dielectric constant of OPV materials, photoexcitation generates strongly bound singlet excitons, which must be dissociated to yield free charges. In a photovoltaic blend, this is achieved by combining two materials in a nanoscale bulk-heterojunction morphology where the difference in energy levels between donor and acceptor components drives exciton dissociation^9^,^10^,^11^. This creates weakly bound CT states in which electron and hole are spatially separated but retain a Coulombic binding (Fig. 1a). These states, which may also be formed through bimolecular recombination, form a crucial step in energy harvesting since their production can limit free charge generation^3^,^12^. Critically, recombination of CT states depends on their spin configuration, and hence they act as an important system in which to explore the spin-dependent processes ubiquitous to organic solar cells, and organic semiconductors in general.

Magnetic resonance techniques naturally lend themselves to probing spin-dependent pathways since they allow for direct spin manipulation. So far, electrically and optically detected magnetic resonance have been the predominant implementations of such techniques, in which changes due to magnetic resonance are monitored through device current, or an optical property such as photoluminescence or absorption^13^,^14^,^15^,^16^,^17^,^18^,^19^,^20^. While these techniques have proven powerful, the possibility of using the dielectric polarizability of photoexcitations to directly probe their spin-dependent recombination has been largely unexplored. Polarizability is an important quantity governing the properties of electronic states in organic solar cells^4^,^21^,^22^, and opens up a novel way of probing spin-selective recombination in a contactless way, without being restricted to studying emissive states only. In this article, we demonstrate this capability and use it to quantitatively investigate spin-dependent pathways in a model system consisting of weakly bound electron-hole pairs recombining at a donor-acceptor interface. By coupling an archetypal polymer:fullerene blend to a high quality-factor microwave resonator, we are able to dielectrically interrogate the spin-dependent recombination of CT states formed directly after exciton dissociation. Using the high microwave magnetic fields that this technique naturally affords, we explore the transition to a regime in which the usual mixing of singlet and triplet CT states due to hyperfine interactions between electron and nuclear spins is dynamically suppressed by the microwave field^23^ manifesting as a Rabi splitting in the population response and revealing coherent spin manipulation in the steady state. We develop a quantitative model to describe this behaviour, and use it to estimate the difference in recombination rate between spin-singlet and spin-triplet CT states. Finally, using this prototypical example of spin-dependent recombination, we highlight the parallels between our experiments conducted on disordered organic blends and the Majorana–Brossel resonances observed in atomic physics experiments on mercury vapour^24^,^25^.

## Results

### Dielectric detection of spin-dependent recombination

Our experimental detection scheme exploits the fact that photoexcitations in an OPV blend result in an increase in the dielectric polarizability of the material relative to the ground state, since they can be more easily polarized^26^. The polarizability can therefore be used as a direct measure of population changes due to spin-dependent recombination: by driving magnetic resonance, a redistribution of spin states causes a net population change due to the spin-selective nature of recombination, which is directly reflected in a change in dielectric polarizability. It is this dielectric detection scheme that we use as a novel probe of spin-dependent recombination.

**Figure 1:**
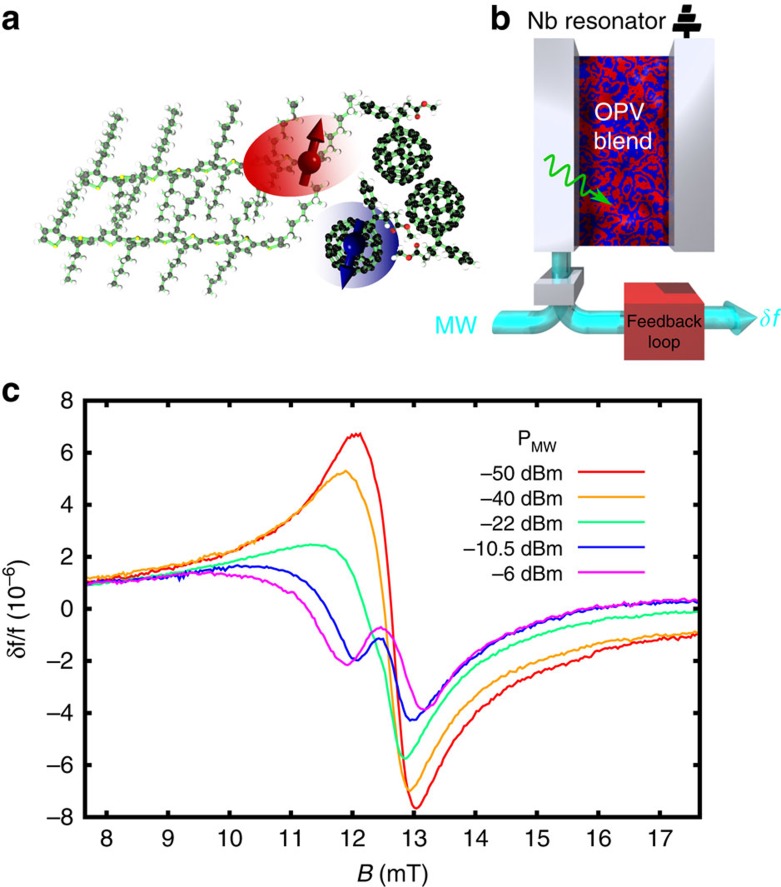
Spin-selective recombination in a photovoltaic blend. (**a**) Illustration of a charge-transfer state consisting of a weakly bound electron (blue) and hole (red), formed across the interface between the polymer P3HT and the fullerene PCBM. (**b**) Simplified schematic of the experimental set-up used to detect spin-dependent recombination through the dielectric polarizability. (In the actual resonator, the Nb lines are meandered.) (**c**) Frequency shift *δf*/*f* of the P3HT:PCBM organic blend coupled to the Nb resonator as a function of magnetic field *B*, and microwave power *P*_MW_.

As a sensitive means to detect changes in dielectric polarizability, we track the shift in resonance frequency of a high-quality factor microwave resonator coupled to our OPV blend under optical illumination (Fig. 1b, and Methods). An increase in excitation density, and hence polarizability, results in the capacitance of the film to be increased, resulting in a drop in the resonance frequency of the resonator 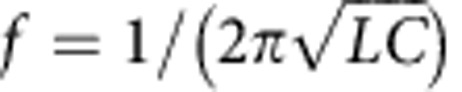, where *C* is the capacitance of the resonator and *L* the inductance. The microwave magnetic and electric fields supplied by the resonator, in conjunction with an external dc magnetic field *B*, allow us to drive spin resonance and track the changes in polarizability through the shift in resonance frequency *δf*. This provides our experimental observable to probe spin-dependent recombination through the dielectric polarizability. As the archetypal polymer:fullerene system, here we use a poly (3-hexyl thiophene)/Phenyl-C61-butyric acid methyl ester) blend (see Methods for further details).

### Spin-dependent recombination among charge-transfer states

**Figure 2:**
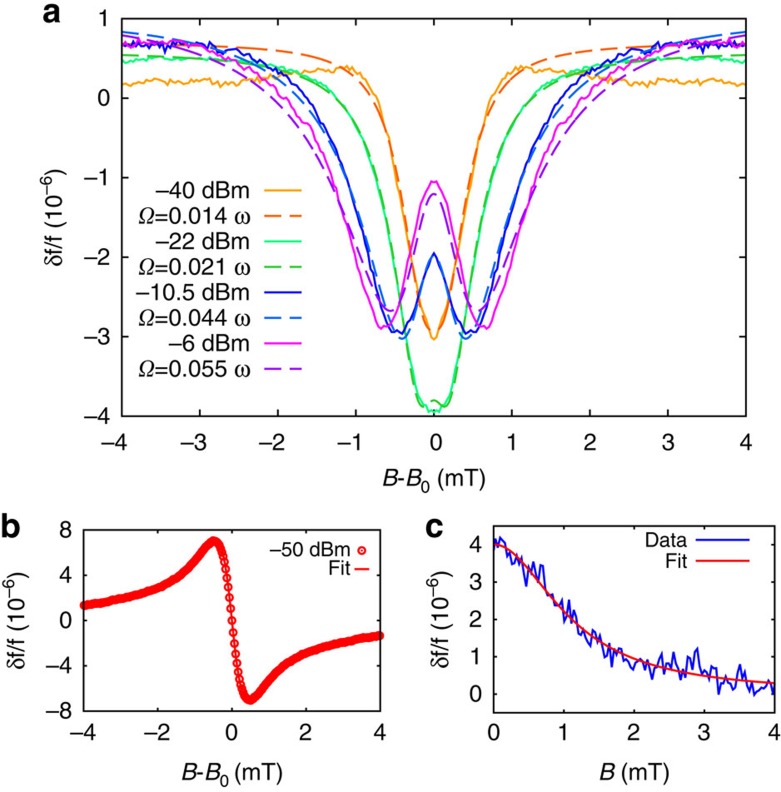
CT state recombination under magnetic resonance. (**a**) Solid lines: frequency shift due to changes in the CT state population under spin resonance. The frequency shift exclusively due to spin-dependent recombination among CT states has been extracted by symmetrising the raw experimental data. Dashed lines: fits to the spin-dependent recombination model, along with the extracted Rabi frequencies *Ω*. (**b**) Low power data, which has been antisymmetrised to extract only the response proportional to the magnetic susceptibility of the blend, along with a fit to the real part of the spin-1/2 Bloch susceptibility *χ*′. (**c**) Low-field response used to estimate the hyperfine field *b*_HF_ which arises from the magnetic interaction between *π*-electron spins and hydrogen nuclear spins.

Figure 1c shows the frequency shift *δf*/*f* of our resonator-OPV blend system as a function of magnetic field and for various incident microwave powers *P*_MW_. At the lowest microwave powers we observe the response expected for a system which contains exclusively free charges–an antisymmetric Lorentzian lineshape proportional to the real part of the spin-1/2 Bloch susceptibility *χ*′ (ref. 27) (Fig. 2b). (We emphasise that this is a contribution to the frequency shift from the magnetic susceptibility of the OPV blend, distinct from the dielectric contribution, which acts as our probe of spin-dependent recombination, as we discuss below.) With increasing microwave power, a qualitatively new behaviour emerges–the lineshape becomes increasingly symmetric and develops two minima whose separation grows with the microwave power. This behaviour cannot be described by the usual Bloch equations for the magnetic susceptibility of a single spin-1/2 carrier which retain an antisymmetric lineshape irrespective of the microwave power^27^. (We confirmed this by performing similar experiments on a reference paramagnetic, di(phenyl)-(2,4,6-trinitrophenyl)iminoazanium). However, as we develop below, the symmetric contribution to the lineshape is instead due to changes in dielectric polarizability from spin-dependent recombination among CT states.

In a photovoltaic blend, we expect a spin-resonance response not only from free charges, but also from bound electron-hole pairs whose population can be altered under magnetic resonance, causing a change in the dielectric polarizability of the OPV film. The two contributions can be clearly seen in the differential expression for the frequency shift


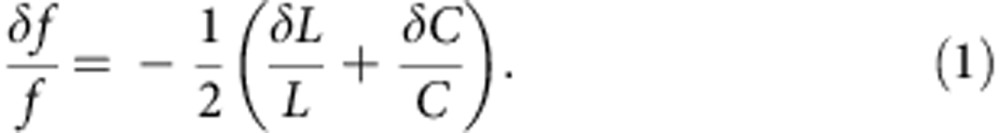


The first term on the right-hand side is proportional to the magnetic susceptibility of the cavity *χ*′, which gives the response that would be obtained in an electron spin resonance experiment (and is proportional to the net magnetization), while the second term is proportional to the dielectric polarizability, and is sensitive to the total population of photoexcitations through their influence on the capacitance^26^,^28^. For a system with purely free charges, only the first term will contribute to the spin-resonant frequency shift, giving a lineshape proportional to the magnetic susceptibility *χ*′, as in Fig. 2b. However, with spin-dependent recombination between oppositely charged carriers, their population can be altered under magnetic resonance, giving rise to a frequency shift due to the change in dielectric polarizability.

We can cleanly separate the frequency shift due to spin-dependent recombination from the magnetic susceptibility response since these have opposite symmetries–the dielectric polarizability response (*δC*/*C*) is symmetric with respect to the line centre, while the magnetic susceptibility (*δL*/*L*) response is antisymmetric. By decomposing our data into symmetric, *S*(*x*)=(*F*(*x*)+*F*(−*x*))/2, and antisymmetric, *A*(*x*)=(*F*(*x*)−*F*(−*x*))/2, data sets where *F*(*x*) is the raw data of Fig. 1, and *x*=*B*−*B*_0_ is the distance from the line centre at *B*_0_, we clearly isolate the dielectric response due to CT state recombination and its evolution with microwave power (Fig. 2a). With increasing microwave power, the peak response develops non-monotonically^23^ and progresses from a lineshape with a single minimum to a split, ‘W'-shaped lineshape with two minima whose separation grows with increasing microwave power. The sign of this dielectric response (*δf*<0) allows us to assign it to spin-dependent recombination within geminate CT states^29^, that is, those which are formed immediately following exciton dissociation. Due to spin conservation, geminate CT states will have spins, which are in either of the antiparallel configurations |↑↓〉, |↓↑〉 (these product states are the energy eigenstates for weakly interacting CT states in the presence of different hyperfine environments). Under spin resonance, these states can be converted to the spin-parallel triplet states |*T*_+_〉=|↑↑〉, |*T*_−_〉=|↓↓〉, which cannot recombine directly to the ground state due to spin-selection rules. This transfer of population to longer-lived states results in an increase in CT-state population, which manifests as a drop in the resonant frequency *δf*<0 due to the increased dielectric polarizability of the film. We note that for CT states formed through bimolecular recombination, the relative populations of singlet and triplet states would be inverted, and we would therefore obtain a frequency shift with the opposite sign. In addition, since geminate CT states are formed from a spin-zero singlet exciton, they do not contribute to the net magnetization, and hence are cleanly decoupled from the antisymmetric magnetic susceptibility response.

### Dynamical suppression of hyperfine mixing

**Figure 3:**
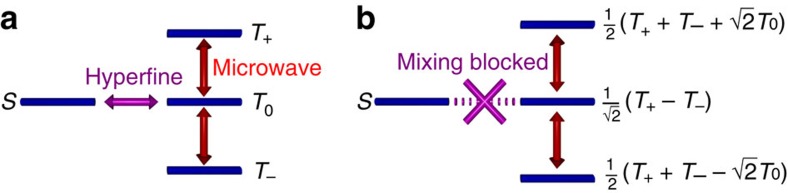
Magnetic resonance among CT states at weak and strong microwave driving. (**a**) For weak driving, the hyperfine-induced mixing between singlet (*S*) and triplet (*T*) levels allows for population changes in the singlet state to be induced. (**b**) For strong microwave driving, a new set of eigenstates emerge which exhibit no hyperfine coupling between singlet and triplet manifolds. This isolates the singlet state, leading to the lineshape splitting seen experimentally.

The high a.c. magnetic fields that our technique naturally offers allows us to explore the transition to a regime where the usual hyperfine-induced mixing between singlet and triplet manifolds can be suppressed (Fig. 3). Off resonance, or for low Rabi frequencies *Ω*=*gμ*_B_*B*_ac_/*ℏ–*where *B*_ac_ is the ac magnetic field strength, *g* is the electron *g*–factor and *μ*_B_ the Bohr magneton–the eigenstates of the spin pair are the simple product states since the difference in hyperfine fields between the two spins results in effective mixing of the singlet state 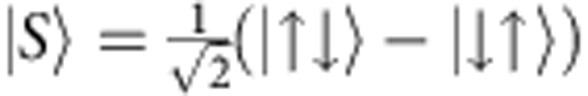 and the 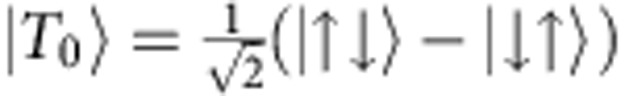 triplet state—Fig. 3a. Microwave transitions can therefore induce a change in the singlet population, and hence a net population change, which we detect through the dielectric polarizability. For sufficiently high Rabi frequencies and on-resonance, a distinct regime emerges where the CT state eigenstates are altered, becoming states with definite spin multiplicity—Fig. 3b. Crucially, a new effective |*T*_0_〉 state 
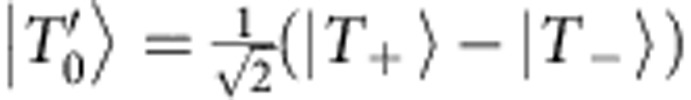
 emerges consisting of a coherent superposition of the off-resonance eigenstates. The creation of this new superposition state blocks mixing of the singlet and triplet manifolds since there are no matrix elements connecting the 
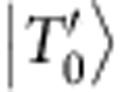
 and |*S*〉 wavefunctions via the difference in hyperfine fields^23^. The singlet state therefore becomes dynamically isolated from the triplet states, and hence no population change of the singlet state can occur (further details are contained in Supplementary Note 1). The population response thus returns to its off-resonance value, resulting in the central dip towards zero in the lineshape. In addition, this lineshape splitting grows with increasing Rabi frequency since this governs how far off resonance it is necessary to go to recover the mixing between singlet and triplet manifolds. We note that the splitting we observe here occurs in a similar regime as the spin locking behaviour observed in pulsed magnetic resonance experiments that is connected with the onset of Rabi oscillations at frequency 2*Ω* (refs 30, 31, 32, 33). While these experiments demonstrate the joint precession of electron and hole spins, here we use our novel detection scheme as a direct probe of the steady state wavefunctions and in particular, the dynamical isolation of the 
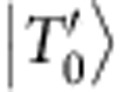
 state.

### Quantitative model of spin-dependent recombination

To construct a detailed picture of this process, we consider a system of CT states which are generated optically, that is, in either of the spin antiparallel configurations, and undergo spin-dependent recombination under the influence of microwave driving. Since electron and hole are spatially separated in a weakly bound CT state they can, to a good approximation, be taken as non-interacting, but we allow for each spin to experience a different hyperfine field, which provides the coupling between the |*S*〉 and |*T*_0_〉 states and as outlined above, plays a crucial role in this phenomenon. To model this system, we work within the rotating frame approximation, and solve the master equation for the CT state density matrix 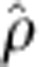 in the presence of microwave driving,


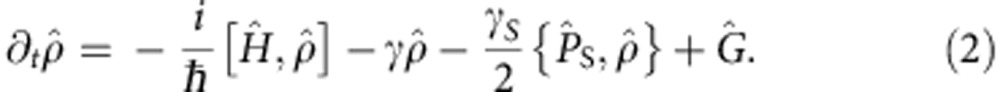


where 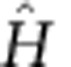 is the (time independent) Hamiltonian consisting of the effective Zeeman and hyperfine interactions for each spin as well as the rotating-frame representation of the microwave field. The square brackets denote the commutator. For simplicity, we use a single spin-independent decay rate γ, but we verified that our conclusions are not significantly altered by including different decay rates for the populations (diagonal density matrix elements), and coherences (off-diagonal elements). Spin-dependent recombination is included through the third term on the right of equation (2) where *γ*_S_ is the spin-dependent annihilation rate, and 
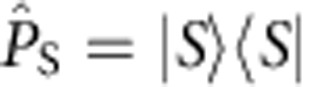
 is the singlet projector. The curly braces denote the anticommutator. Finally, 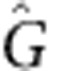 is the source term describing generation of geminate CT states, that is, states created in one of the two antiparallel spin configurations. The change in CT state population due to microwave driving is then 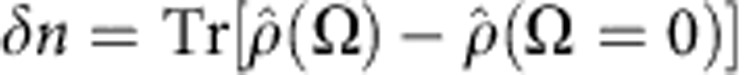, where 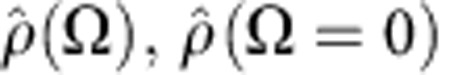 are the responses with and without the ac magnetic field. This results in a frequency shift *δf*/*f*=−*αδn*, where α is the coupling constant between photoexcitation number and frequency shift, and is determined by the dielectric polarizability of the CT states^26^. Following the analytic procedure outlined in the Methods section, we derive the following expression for the frequency shift


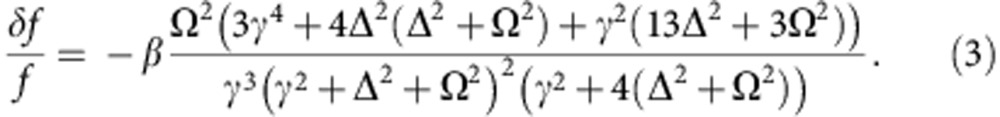


Here *β*=2*Nαγ*_S_*ω*_HF_^2^, where *N* is the population of geminate CT states, *ω*_HF_=*gμ*_B_*b*_HF_/*ℏ* where 2*b*_HF_ is the difference in hyperfine fields between the two spins, and Δ=*ω*_0_−*ω* with *ω*_0_=*gμ*_B_*B*/*ℏ* and *ω*=2*πf*. As an upper bound for the spin-independent decay rate, we fix *γ*=1/13 ns^−1^ from the linewidth of the low power data (Fig. 2b). The only remaining fit parameters are the Rabi frequency, and the constant pre-factor β. We fix this across the whole data set, leaving only the Rabi frequency *Ω* to vary. The resulting fits are shown in Fig. 2a, and show excellent agreement with the experimental data over the range of powers used.

Our experiments allow us to simultaneously estimate the hyperfine field *b*_HF_ from the static-field response of the organic blend at low magnetic fields (Fig. 2c). This behaviour, which has been described in detail in the context of magnetic field effects in organic devices^34^,^35^,^36^,^37^,^38^, results from the lifting of the hyperfine-induced spin mixing for the |*T*_+_〉,|*T*_−_〉 triplet states when the external field becomes comparable to the hyperfine field, and hence provides an estimate of *b*_HF_. Fitting this low-field response to a Lorentzian lineshape, we find a half-width half-maximum of *b*_HF_=1.1 mT, consistent with other estimates of the hyperfine field in OPV materials^32^,^39^. If we also assume that the total excitation density is of the same order as the geminate CT state density, we can also use the change in resonance frequency on illumination at *B*=0, Δ*f*/*f*, to estimate *N* from Δ*f*/*f*=2 × 10^−4^∼−*Nα*, which leaves *γ*_S_ as the only undetermined parameter in the fits in Fig. 2. Using the parameters above, we find *γ*_S_≈1 μs^−1^ that provides an estimate for the difference in recombination rate between singlet and triplet CT states. This an important parameter to be able to quantify to understand spin-dependent recombination processes in organic semiconductors, and can otherwise be challenging to determine.

**Figure 4:**
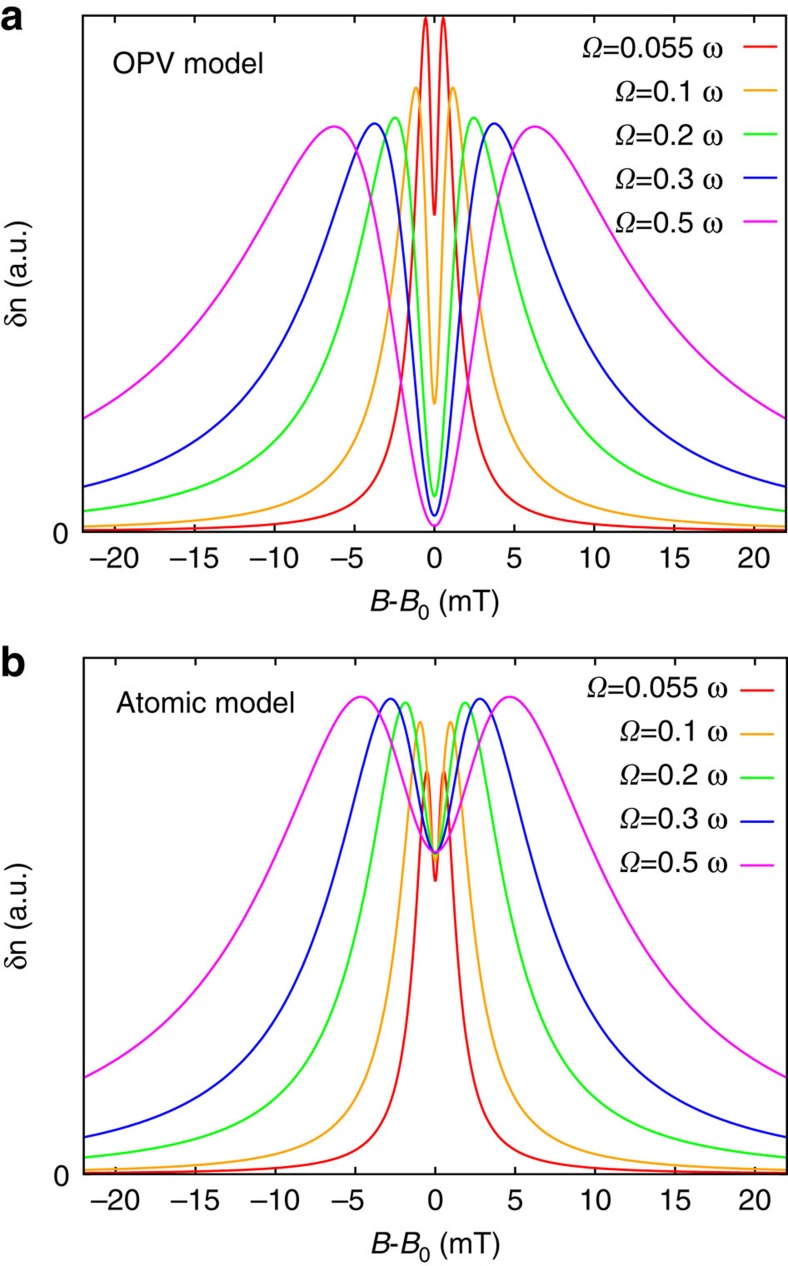
Comparison with Majorana–Brossel resonances. Simulated microwave-induced population changes *δn* for different Rabi frequencies *Ω* for (**a**) charge-transfer states in a photovoltaic (OPV) blend and (**b**) the *m*=0 triplet sublevel in mercury vapour.

Finally, it is interesting to compare the behaviour observed here in organic semiconductor materials with the Majorana–Brossel resonances obtained in atomic physics experiments on mercury vapour^24^, where the change in population of the *m*=0 triplet excited state was monitored through optical depolarization. In those experiments, linearly polarized radiation was used to selectively populate the *m*=0 triplet sublevel, and microwave transitions to the *m*=±1 states altered the polarization of the emitted radiation, giving an effective measure of the *m*=0 sublevel population. This probing of spin-resonant population changes of the *m*=0 state has clear parallels with our experiments which probe the spin-singlet content of an electron-hole pair, despite the two phenomena occurring in systems with very different spin environments. In the atomic physics case, a lineshape splitting developed when the Rabi frequency became larger than the excited state decay rate and the separation between peaks in the split lineshape was proportional to the Rabi frequency *Ω*, revealing coherent spin manipulation in steady state measurements, as we also observe here. Figure 4 compares the lineshapes obtained from equation (3) with those derived in the atomic physics experiments^24^,^25^ (see Methods), where we have used the same Rabi frequencies, and have taken the mean excited state lifetime of mercury atoms as *γ*^−1^. The Rabi splitting is much clearer in the OPV case than for the atomic case: for the OPV, the population response falls to zero at the centre of the pattern at sufficiently high driving, while in the atomic case the amplitude drops by a maximum of 1/3 of its peak value. This highlights the strict dynamical isolation of the singlet state possible in the four-level OPV system due to the coherent generation of the 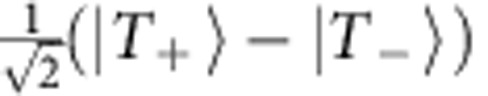 state.

## Discussion

The rich spin-dependent phenomena in organic semiconductors are both crucial for the understanding and implementation of optoelectronic and spintronic devices, while simultaneously providing a wealth of possibilities to explore the physics of spins residing in a disordered environment. By using the dielectric polarizability to probe spin-dependent recombination in an organic solar cell blend, we have presented a new way of exploring both of these possibilites. Through this dielectrically detected magnetic resonance technique we have been able to estimate the spin-dependent recombination rate among charge-transfer states formed in a photovoltaic blend. Furthermore, we have used this technique to observe the dynamical suppression of hyperfine mixing between singlet and triplet manifolds through the creation of a coherent blocking mode. Unlike techniques using optical detection, the method introduced here is not limited to emissive states, and can therefore be used to investigate non-emissive spin-selective recombination channels. It is also contactless, allowing direct probing of bound states, without relying on their subsequent impact on electrical current, as well as simplifying sample preparation. The use of a new macroscopic observable opens up future avenues to explore and quantify the spin-dependent pathways intrinsic to organic semiconductors.

## Methods

### Experimental set-up

To measure spin-dependent recombination through the dielectric polarizability, an OPV blend is spin-coated on a high-quality factor (*Q*≃10^5^) Nb resonator, which is mounted inside an optically accessible cryostat magnet providing a dc magnetic field *B*. The sample is held at a temperature of *T*=2 K, and illuminated with a 532 nm laser providing an intensity of ∼100 mW cm^−2^. The resonator is operated at its fundamental frequency of *f*≃365 MHz by driving with a microwave source. This creates a.c. magnetic and electric fields across the film, which allow us to drive spin resonance and measure the dielectric polarizability, respectively. A microwave reflectrometry circuit in conjunction with a feedback loop is used to monitor the resonance frequency as the static magnetic field is swept, giving the frequency shift due to spin resonance transitions (after subtraction of an illumination-independent parabolic response^40^). We note that it is the real part of the susceptibility that is measured in our experiments since we detect the resonance frequency shift, rather than the cavity dissipation. Here we use a 1:1 weight ratio poly (3-hexyl thiophene)/Phenyl-C61-butyric acid methyl ester) blend, prepared from a chlorobenzene solution and annealed at 130 ^°^C under nitrogen atmosphere. Reference experiments were performed on the paramagnet di(phenyl)-(2,4,6-trinitrophenyl)iminoazanium that contains isolated spins only.

### Simulations

For weakly interacting CT states, the static Hamiltonian is diagonal in the product basis of the two individual spins, and the time dependence of the full Hamiltonian can be removed by making a transformation to the rotating frame, leading to equation (2). To proceed analytically, we treat the spin-dependent recombination term as a perturbation on the spin-independent relaxation, and introduce equal and opposite hyperfine fields ±*b*_HF_ for each spin. (We note that we also numerically solved equation (2), averaging over Gaussian hyperfine distributions for each spin, and found good agreement with the simpler analytic treatment presented here. Examples of the numerical simulations are shown in Supplementary Fig. 1 and further details are contained in Supplementary Note 2). Making one more unitary transformation which diagonalizes 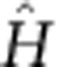, we expand to leading order in *ω*_HF_=*gμ*_B_*b*_HF_/*ℏ* to obtain equation (3) as the steady state solution of the master equation (equation (2)).

### Majorana–Brossel resonances

In Fig. 4b, we use equation (5) from ref. 24:


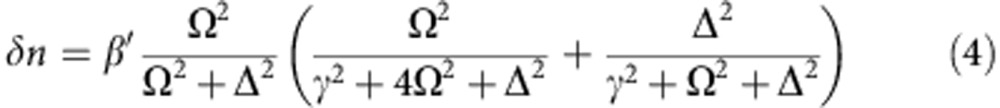


where *β*′ is a constant and we have used *γ*^−1^ for the mean excited state lifetime.

### Data deposition

The data underlying this paper are available at www.repository.cam.ac.uk/handle/1810/249110

## Additional information

**How to cite this article:** Bayliss, S. L. *et al*. Spin-dependent recombination probed through the dielectric polarizability. *Nat. Commun.* 6:8534 doi: 10.1038/ncomms9534 (2015).

## Supplementary Material

Supplementary Information Supplementary Figure 1, Supplementary Notes 1-2 and Supplementary References
