# Supplementary material for: Spin-dependent recombination probed through the dielectric polarizability
Source: Nat Commun. 2015 Oct 6;6:8534. doi: 10.1038/ncomms9534 (PMC4600752; doi:10.1038/ncomms9534)
Supplement: Supplementary Information — Supplementary Figure 1, Supplementary Notes 1-2 and Supplementary References [file ncomms9534-s1.pdf]

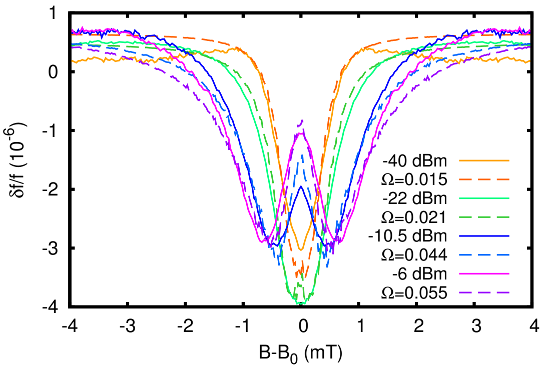

Supplementary Figure 1: **Numerical simulations of Rabi splitting.** Simulated frequency shifts (dotted lines) for different Rabi frequencies Ω (in units of the microwave frequency ω) using $\gamma = 0.035\,\omega$ (which corresponds to the linewidth determined from the antisymmetrised experimental data), a standard deviation of hyperfine fields of $\sigma_{\mathrm{HF}} = 0.01\,\omega$, and $\gamma_{\mathrm{S}} = \gamma/100$. The experimental data (solid lines) at different microwave powers is shown for comparison.

Supplementary Note 1: Dynamical Supression of Hyperfine Mixing

For low Rabi frequencies, or for sufficient detuning from resonance, the eigenstates of the static Hamiltonian are the product states

$$\begin{aligned}
|\phi_1\rangle &= |\uparrow\uparrow\rangle = |T_+\rangle \\
|\phi_2\rangle &= |\uparrow\downarrow\rangle = \frac{1}{\sqrt{2}}(|S\rangle + |T_0\rangle) \\
|\phi_3\rangle &= |\downarrow\uparrow\rangle = \frac{1}{\sqrt{2}}(|T_0\rangle - |S\rangle) \\
|\phi_4\rangle &= |\downarrow\downarrow\rangle = |T_-\rangle
\end{aligned}$$

The two mixed states $|\phi_2\rangle, |\phi_3\rangle$, which do not have definite spin multiplicity, are connected directly with the pure triplet states $|\phi_1\rangle, |\phi_4\rangle$ by microwave transitions e.g. $\langle\phi_4| \hat{S}_x |\phi_2\rangle \neq 0$ where $\hat{S}_x = \hat{\sigma}_x \otimes I + I \otimes \hat{\sigma}_x$ with $\hat{\sigma}_x$ the x-component Pauli spin matrix, and the x-direction is perpendicular to the static field. In this case, population changes in the singlet state can be induced directly, giving rise to a change in CT-state population due to spin-dependent recombination.

Alternatively, we can consider the coupling between $|S\rangle$ and $|T_0\rangle$ states induced by the difference in hyperfine fields $|\langle S| \hat{H}_{\mathrm{HF}} |T_0\rangle|$where

$$\hat{H}_{\mathrm{HF}} = \hat{h}_{\mathrm{HF}}(\omega_{\mathrm{HF}}) \otimes I + I \otimes \hat{h}_{\mathrm{HF}}(-\omega_{\mathrm{HF}})$$

and

$$\hat{h}_{\mathrm{HF}}(\omega_{\mathrm{HF}}) = \begin{pmatrix} \omega_{\mathrm{HF}} & 0 \\ 0 & -\omega_{\mathrm{HF}} \end{pmatrix}. \tag{1}$$

We find $|\langle S| \hat{H}_{\mathrm{HF}} |T_0\rangle| = \hbar\omega_{\mathrm{HF}}$ and hence the (degenerate) $|S\rangle$ and $|T_0\rangle$ states are effectively mixed, allowing microwave-induced population changes to be reflected in the singlet population.

For sufficiently high microwave driving and near resonance, where the Rabi frequency dominates over the hyperfine field ω_{HF}, the decay rate γ and the detuning Δ, the single spin Hamiltonians in the rotating frame can be approximated as

$$\hat{h}^{(e/h)} \approx \frac{\hbar}{2} \begin{pmatrix} 0 & \Omega \\ \Omega & 0 \end{pmatrix} \tag{2}$$

where e/h denote electron and hole respectively. This Hamiltonian has eigenvectors $\frac{1}{\sqrt{2}}(|\uparrow\rangle \pm |\downarrow\rangle)$ and in this regime the eigenstates become

$$\begin{aligned}
|\psi_1\rangle &= \frac{1}{2}(|T_+\rangle + |T_-\rangle + \sqrt{2}\,|T_0\rangle) \\
|\psi_2\rangle &= |S\rangle \\
|\psi_3\rangle &= \frac{1}{\sqrt{2}}(|T_+\rangle - |T_-\rangle) \\
|\psi_4\rangle &= \frac{1}{2}(|T_+\rangle + |T_-\rangle - \sqrt{2}\,|T_0\rangle)
\end{aligned}$$

These states now all have a definite spin multiplicity. Furthermore, there is no longer any hyperfine-induced coupling between the singlet state $|\psi_2\rangle$ and the (degenerate) $|\psi_3\rangle$ state since $\langle\psi_2|\hat{H}_{\mathrm{HF}}|\psi_3\rangle = 0$ [1]. With the formation of this new coherent state $\frac{1}{\sqrt{2}}(|T_+\rangle - |T_-\rangle)$, the singlet state is therefore isolated from the triplet manifold since there are no matrix elements connecting the singlet states to the other three states, and so the population change approaches zero.

Upon detuning, the coupling between singlet and triplet manifolds is recovered, which we can see by setting $\Delta^{(\mathrm{e/h})} = \Omega$ so that

$$\hat{h}^{(\mathrm{e/h})} \approx \frac{\hbar}{2}\begin{pmatrix}\Omega & \Omega \\ \Omega & \Omega\end{pmatrix} \tag{3}$$

In this case, the two degenerate eigenstates are

$$\begin{aligned} |\Psi_2\rangle &= \frac{1}{\sqrt{6}}(|T_+\rangle - |T_-\rangle - \sqrt{2}\,|S\rangle - \sqrt{2}\,|T_0\rangle) \\ |\Psi_3\rangle &= \frac{1}{2\sqrt{3}}(|T_+\rangle - |T_-\rangle + 2\sqrt{2}\,|S\rangle - \sqrt{2}\,|T_0\rangle) \end{aligned}$$

The coupling between these two states due to the hyperfine interaction is $|\langle\Psi_2|\hat{H}_{\mathrm{HF}}|\Psi_3\rangle| = \frac{\sqrt{2}}{3}\hbar\omega_{\mathrm{HF}}$, restoring the mixing between singlet and triplet manifolds, and hence the microwave-induced population change.

Supplementary Note 2: Numerical Simulations

To complement the analytic treatment, we also performed numerical simulations of equation (2) in the main text. Working in the 4×4 product basis of the two spins $\{|\uparrow\uparrow\rangle, \uparrow\downarrow, |\downarrow\uparrow\rangle, |\downarrow\downarrow\rangle\}$ the explicit representations of the relevant terms in equation (2) are given by

$$\hat{H} = \hat{h}^{(\mathrm{e})} \otimes I + I \otimes \hat{h}^{(\mathrm{h})} \tag{4}$$

where

$$\hat{h}^{(\mathrm{e})} = \frac{\hbar}{2}\begin{pmatrix}\Delta^{(\mathrm{e})} & \Omega \\ \Omega & -\Delta^{(\mathrm{e})}\end{pmatrix} \tag{5}$$

and likewise for $\hat{h}^{(\mathrm{h})}$. Here I is the 2×2 identity matrix, and the hyperfine field features in the detuning between electron and hole: $\Delta^{(\mathrm{e})} - \Delta^{(\mathrm{h})} = 2\omega_{\mathrm{HF}}$. In its matrix representation, the singlet projector is given by

$$\hat{P}_{\mathrm{S}} = |S\rangle\langle S| = \frac{1}{2}\begin{pmatrix}0 & 0 & 0 & 0 \\ 0 & 1 & -1 & 0 \\ 0 & -1 & 1 & 0 \\ 0 & 0 & 0 & 0\end{pmatrix}. \tag{6}$$

The generation term, which represents creation of electron-hole pairs in one of the antiparallel energy eigenstates is given by

$$\hat{G} = \frac{1}{2}(|S\rangle\langle S| + |T_0\rangle\langle T_0|) = \frac{1}{2}\begin{pmatrix}0 & 0 & 0 & 0 \\ 0 & 1 & 0 & 0 \\ 0 & 0 & 1 & 0 \\ 0 & 0 & 0 & 0\end{pmatrix}. \tag{7}$$

For computational convenience we rewrite the 4×4 density matrix of equation (2) as a 16×1 column vector, and the homogeneous terms as 16×16 superoperators, which we denote by tildes:

$$\partial_t\hat{\rho} = -(\frac{i}{\hbar}\tilde{H} + \tilde{\gamma} + \frac{\gamma_{\mathrm{S}}}{2}\tilde{P}_{\mathrm{S}})\hat{\rho} + \hat{G} \tag{8}$$

Here $\tilde{H}$, and $\tilde{P}_{\mathrm{S}}$ are the superoperator representations of the commutator $[\hat{H}, \hat{\rho}]$ and the anticommutator $\{\hat{P}_{\mathrm{S}}, \hat{\rho}\}$, and are given by

$$\begin{aligned} \tilde{H} &= \hat{H}\otimes\mathbb{I} - \mathbb{I}\otimes\hat{H} \\ \tilde{P}_{\mathrm{S}} &= \hat{P}_{\mathrm{S}}\otimes\mathbb{I} + \mathbb{I}\otimes\hat{P}_{\mathrm{S}} \end{aligned}$$

where $\mathbb{I}$ is the 4×4 identity matrix [2]. The spin-independent decay term becomes a diagonal 16×16 matrix, and $\hat{G}$ is now a 16×1 column vector. In compact form, we have

$$\tilde{L}\hat{\rho} = \hat{G} \tag{9}$$

where $\tilde{L} = \frac{i}{\hbar}\tilde{H} + \tilde{\gamma} + \frac{\gamma_S}{2}\tilde{P}_{\mathrm{S}}$ and hence

$$\hat{\rho} = \tilde{L}^{-1}\hat{G} \tag{10}$$

The problem is therefore reduced to a matrix inversion of the 16×16 matrix $\tilde{L}$. The population change as a function of magnetic field is then

$$\begin{aligned}\delta n(B) &= \mathrm{Tr}[\hat{\rho}(B)] \\ &= \mathrm{Tr}[\tilde{L}^{-1}(B)\hat{G}]\end{aligned}$$

We also include the role of the hyperfine interaction by adding a random hyperfine field to the Hamiltonian of each spin, which we draw from separate Gaussian distributions with equal standard deviation σ_{HF}. For each value of the magnetic field, we average over several thousand hyperfine fields to obtain the resulting population response of Supplementary Figure 1 which shows good agreement with the analytic calculations.

Supplementary References

[1] Roundy, R. C. & Raikh, M. E. Organic magnetoresistance under resonant ac drive. *Phys. Rev. B* **88**, 125206 (2013).

[2] Lancaster, P. Explicit solutions of linear matrix equations. *Siam Rev.* **12**, 544–566 (1970).
